# Acute compartment syndrome in an anterior cruciate ligament revision: a case report

**DOI:** 10.1093/jscr/rjaf916

**Published:** 2025-11-19

**Authors:** Bujar Shabani, Dijon Musliu, Vlora Podvorica, Dafina Bytyqi

**Affiliations:** Clinic of Orthopedics, University Clinical Center of Kosovo, Prishtina, Kosovo, Albania; Faculty of Medicine, University of Prishtina “Hasan Prishtina”, Prishtina, Kosovo, Albania; Clinic of Orthopedics, University Clinical Center of Kosovo, Prishtina, Kosovo, Albania; Faculty of Medicine, University of Prishtina “Hasan Prishtina”, Prishtina, Kosovo, Albania; Clinic of Orthopedics, University Clinical Center of Kosovo, Prishtina, Kosovo, Albania; Clinic of Orthopedics, University Clinical Center of Kosovo, Prishtina, Kosovo, Albania; Faculty of Medicine, University of Prishtina “Hasan Prishtina”, Prishtina, Kosovo, Albania

**Keywords:** acute compartment syndrome, ACL revision, case report

## Abstract

Acute compartment syndrome (ACS) is a rare but serious complication of anterior cruciate ligament (ACL) reconstruction. We present the case of a 21-year-old male soccer player who developed ACS during revision ACL reconstruction. The patient had previously undergone ACL reconstruction and revision was indicated for recurrent instability and graft rupture confirmed by MRI. At the end of the procedure, marked intraoperative edema prompted a medial fasciotomy, which decompressed the superficial and deep posterior compartments. The patient recovered without complications, returned to sport within nine months, and experienced no residual deficits. This case is distinct in that ACS was diagnosed intraoperatively, enabling immediate fasciotomy and preventing postoperative morbidity. Comparison with existing literature highlights that while ACS after ACL reconstruction is uncommon, it remains a critical risk. Lessons include the need for intraoperative vigilance, timely surgical decompression when swelling suggests impending ACS, and the potential for excellent functional outcomes with early intervention.

## Introduction

Acute compartment syndrome (ACS) is an uncommon but urgent condition that can threaten limb function and, in severe cases, cause death [1]. It results from bleeding or swelling within a closed muscle compartment, which compromises neurovascular structures and tissue viability. Prompt diagnosis and fasciotomy are essential to prevent irreversible damage [2]. The arteriovenous pressure gradient hypothesis suggests that trauma-induced increases in compartment pressure elevate venous pressure, reduce the arteriovenous gradient, and ultimately impair tissue perfusion [3, 4]. Clinically, ‘pain disproportionate to the injury’ is the most important symptom, especially when unrelieved by analgesia, and should raise strong suspicion for ACS [3, 5]. In the intraoperative setting, however, physical examination is limited, as anesthetized patients cannot report hallmark symptoms.

Although trauma accounts for ~70% of ACS cases, it has also been reported after minor injuries, muscle tears, and elective surgeries, including ACL reconstructions as well as total hip and knee arthroplasty [6–9]. With the rising frequency of primary ACL reconstructions, revision procedures are also increasing [10, 11]. Here, we report a case of ACS diagnosed intraoperatively during a revision ACL reconstruction.

## The case

A 21-year-old male soccer player presented with recurrent right knee injury. He had undergone ACL reconstruction with a hamstring tendon graft 2 years earlier and returned to competitive soccer without complaints. The new injury occurred during a match. Examination showed a soft endpoint on the Lachman test and a ++ pivot shift, indicating significant instability. MRI confirmed rupture of the previous graft. CT revealed bone tunnels < 10 mm and incompletely ossified. Based on these findings, a one-stage revision was planned.

The patient underwent 5 weeks of intensive physiotherapy prior to the surgery to optimize knee stability and functionality. The revision surgery involved the implantation of a bone-patellar tendon-bone (BTB) graft combined with lateral extra-articular tenodesis to enhance joint stability. A secondary tether using the iliotibial band was added to augment rotational stability, as recurrent instability is more common in revision cases, particularly in young athletes with high functional demands. The revision surgery was performed under spinal (rachianesthesia) anesthesia. An arthroscopy fluid pump was used to maintain joint distension during the procedure.

The procedure began with knee arthroscopy to assess and prepare the joint, followed by harvesting of the BTB graft. A lateral incision was made to facilitate the iliotibial band graft for the tenodesis.

Notably, the incision for the initial hamstring graft was positioned more medially compared to the more lateral incision used for the BTB graft. At the conclusion of the surgery, significant edema was observed (tourniquet was used only 60 minutes). Compartment pressures were measured intraoperatively and compared with the patient’s diastolic blood pressure to calculate delta pressures. The delta pressure in the anterior compartment was 5 mmHg, in the lateral compartment 16 mmHg, and in the posterior compartment there was a borderline delta pressure that could not be confirmed as compartment syndrome. These findings confirmed the presence of compartment syndrome requiring decompression.

The swelling was identified intraoperatively, while the patient was still under anesthesia. A fasciotomy was performed, which effectively released the superficial and deep posterior compartments. A single lateral incision was selected to minimize morbidity, as the edema was most prominent medially and decompression through this approach was deemed sufficient. A pneumatic tourniquet was applied for 60 minutes during graft harvest and tunnel preparation, which is our standard protocol for revision ACL surgery. The arthroscopy itself was technically demanding due to prior tunnel placement and scar tissue but was completed without intra-articular complications.

Following fasciotomy and closure, compartment pressure was visibly relieved with improved tissue turgor. Once awake postoperatively, the patient reported no pain or neurological deficits, confirming effective decompression.

This case highlights the challenges associated with recurrent ACL injuries and underscores the importance of tailored surgical interventions to manage complex cases effectively, ensuring positive outcomes and facilitating return to athletic performance.

## Discussion

ACS is a rare complication of ACL reconstruction [8, 12, 13]. Most reported cases are diagnosed postoperatively, when patients present with progressive pain, neurological deficits, and tense compartments [8, 12–14]. In contrast, our case is unique because ACS was recognized intraoperatively, before wound closure, allowing immediate fasciotomy and prevention of postoperative morbidity [8, 12, 13].

Kudo *et al*. described ACS 2 days after revision ACL surgery, likely due to injury of collateral vessels during hamstring graft harvesting [12]. Filho *et al*. reported a case where severe pain and neurological deficits developed within ten hours; intracompartmental pressure measured 85 mmHg, and bilateral fasciotomies were required, followed by skin grafting [8]. Takahashi et al. observed ACS 12 hours after surgery, secondary to a tibial tunnel hematoma compressing the medial calf compartment [13]. Kindle et al. reported ACS presenting on postoperative day 4 with erythema, pain, and foot drop, requiring fasciotomies; the patient recovered fully by 6 months [14].

Unlike these delayed presentations, our case was confirmed intraoperatively. Delta pressures were 5 mmHg in the anterior compartment and 16 mmHg in the lateral compartment, both well below the diagnostic threshold of 30 mmHg, consistent with ACS. The posterior compartment showed a borderline delta pressure, but overall findings warranted decompression. A single medial fasciotomy was performed, effectively releasing the superficial and deep posterior compartments. In contrast to reports requiring dual incisions [8, 13], our case highlights the importance of tailoring the surgical approach to intraoperative findings.

The fasciotomy wound healed by secondary intention without grafting. The patient completed structured rehabilitation, regained full motion, returned to running at three months, and resumed competitive soccer at nine months without instability or neurological deficits.

This case underscores three lessons: [1] surgeons must remain alert to ACS even in elective revision ACL surgery; [2] intraoperative recognition of swelling and low delta pressures should prompt timely fasciotomy; and [3] individualized decompression and structured rehabilitation can achieve excellent outcomes.

## Conflict of interest statement

None declared.

## Funding

None declared.
